# Influence of Growth Conditions on Magnetite Nanoparticles Electro-Crystallized in the Presence of Organic Molecules

**DOI:** 10.3390/ijms140510383

**Published:** 2013-05-17

**Authors:** Saba Mosivand, Lorena M. A. Monzon, Iraj Kazeminezhad, J. Michael D. Coey

**Affiliations:** 1 Physics Department, Trinity College, Dublin 2, Ireland; E-Mails: aranzazl@tcd.ie (L.M.A.M.); jcoey@tcd.ie (J.M.D.C.); 2 Physics Department, Faculty of Science, Shahid Chamran University, Ahvaz 61357-43337, Iran; E-Mail: I.Kazeminezhad@scu.ac.ir

**Keywords:** magnetite nanoparticles, thiourea, sodium butanoate, electro-crystallization, magnetization, magnetite stoichiometry

## Abstract

Magnetite nanoparticles were synthesized by electrocrystallization in the presence of thiourea or sodium butanoate as an organic stabilizer. The synthesis was performed in a thermostatic electrochemical cell containing two iron electrodes with an aqueous solution of sodium sulfate as electrolyte. The effects of organic concentration, applied potential and growth temperature on particle size, morphology, structure and magnetic properties were investigated. The magnetite nanoparticles were characterized by X-ray diffraction, electron microscopy, magnetometry and Mössbauer spectrometry. When the synthesis is performed in the presence of sodium butanoate at 60 °C, a paramagnetic ferric salt is obtained as a second phase; it is possible to avoid formation of this phase, increase the specific magnetization and improve the structure of the oxide particles by tuning the growth conditions. Room-temperature magnetization values range from 45 to 90 Am^2^kg^−1^, depending on the particle size, type of surfactant and synthesis conditions. Mössbauer spectra, which were recorded at 290 K for all the samples, are typical of nonstoichiometric Fe_3−δ_O_4_, with a small excess of Fe^3+^, 0.05 ≤ δ ≤ 0.15.

## 1. Introduction

In the last decade, magnetic nanostructures have attracted great attention because of their special properties, which differ significantly from those of the bulk materials. It is well known that magnetic nanoparticles are suitable for many industrial, biological and medical applications in the fields of pharmaceutical and cosmetic products, polishing, coatings, catalysis and semiconductors [1,2]. Recently, they have found new applications in areas such as cell separation and detection [3], inmuno-recognition and detection [4], tissue engineering and regenerative medicine [5], imaging [6], and in biosensors [7,8].

Iron oxide nanoparticles are particularly useful and are being successfully employed in ferrofluids [9], hyperthermia-based therapy, controlled drug delivery [10,11], magnetic resonance imaging [12], drug delivery [13], as well as removal of heavy metals from waste water [14,15]. The magnetic properties have been discussed in several recent reviews [1,2,16–19]. They depend on many factors, including the particle size and shape, chemical composition, the type and density of defects and the interactions of the particles with the their neighbors [17].

To date, a wide range of different methods have been used to synthesize magnetite nanoparticles, which includes thermal decomposition [20], co-precipitation [21,22], ball milling [23], and solvothermal synthesis [24]. Co-precipitation is one of the simplest and oldest techniques and also the most common production method. However, this method usually generates particles with a wide particle size distribution, and may require secondary size selection [25]. Another difficulty in synthesizing Fe_3_O_4_ nanoparticles by chemical co-precipitation is the strong tendency of the particles to oxidize to αFe_2_O_3_, thereby greatly reducing their magnetization. Hence, the reaction should be performed under an inert atmosphere.

Electro-crystallization (electro-oxidation) is a less conventional method for preparing iron oxide nanoparticles. In this method, by applying an appropriate potential difference between two iron plates in an aqueous electrolyte solution, iron at the anode is oxidized to Fe^2+^ and Fe^3+^ and water is reduced to hydrogen and hydroxyl anions at the cathode. The iron ions and OH^−^ in the solution react to form a black magnetite precipitate.

Up to now, there have been only a few reports of electrochemical synthesis of magnetite nanoparticles [26–29]. The mean particle size and particle size distribution can be tuned by growth parameters such as applied potential and temperature. Stabilizer agents can be added to the solution to avoid agglomeration.

It is possible to control the mean particle size and size distribution by adjusting the growth temperature and applied potential, in the presence of tetramethylammonium chloride as a stabilizer agent [26,27]. We have also found that the structural properties of electro-oxidized Fe_3_O_4_ nanoparticles are affected by the stabilizer concentration [29], and that the specific magnetization (σ_s_) of magnetite can be controlled by the capping agent [30]. A great advantage of this synthetic route is that an inert atmosphere is not necessary to avoid oxidation of the nanoparticles.

The present report on the synthesis of magnetite nanoparticles focuses on two different organic molecules: thiourea (Tu) and sodium butanoate (Bu). We systematically examine the effect of concentration of organic additives, magnitude of applied potential and bath temperature on the structural and magnetic properties of the magnetite. We also analyze the relationship between growth conditions, specific magnetization and particle size, which is largely dependent on the concentration of organic molecules.

## 2. Results and Discussion

### 2.1. XRD Results

**Figure 1:**
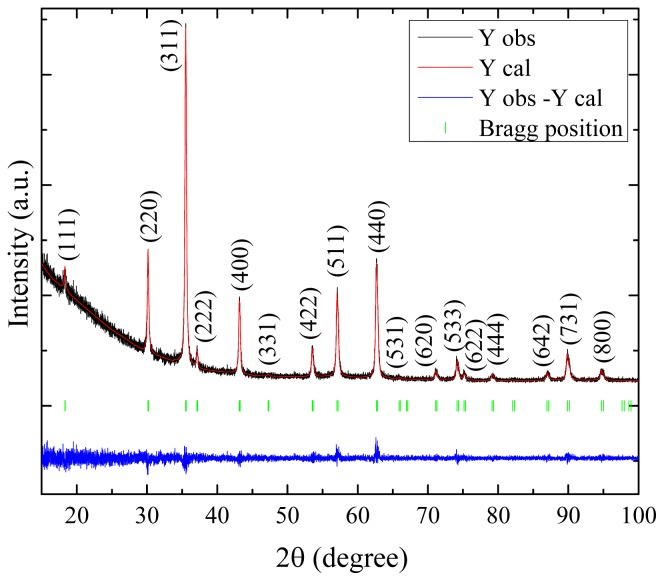
The XRD pattern and the Rietveld profile refinement, using the Fullprof program for magnetite nanoparticles prepared at 60 °C with 0.04 M Tu concentration, applying 5 volts across the electrochemical cell.

A typical XRD pattern with Rietveld analysis of a magnetite nanoparticle sample is shown in Figure 1. Based on XRD results, all products synthesized by this technique have the cubic spinel structure with space group Fd3̄m. The lattice parameters were all in the range 0.8371–0.8403 nm, which may be comparable with the reference value for stoichiometric magnetite of 0.8396 nm.

### 2.2. Particle Size Distribution and Morphology

**Figure 2:**
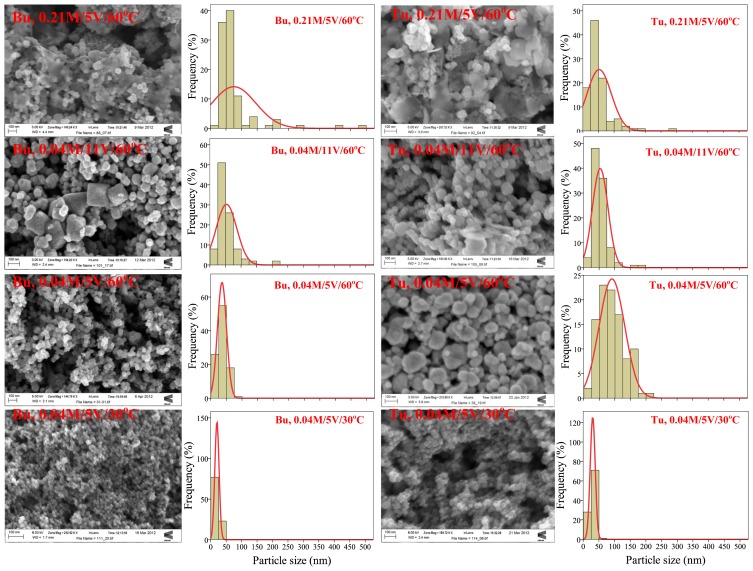
SEM images with size distribution histograms of magnetite nanoparticles prepared at different growth condition in the presence of Bu (**left**) and Tu (**right**).

**Figure 3:**
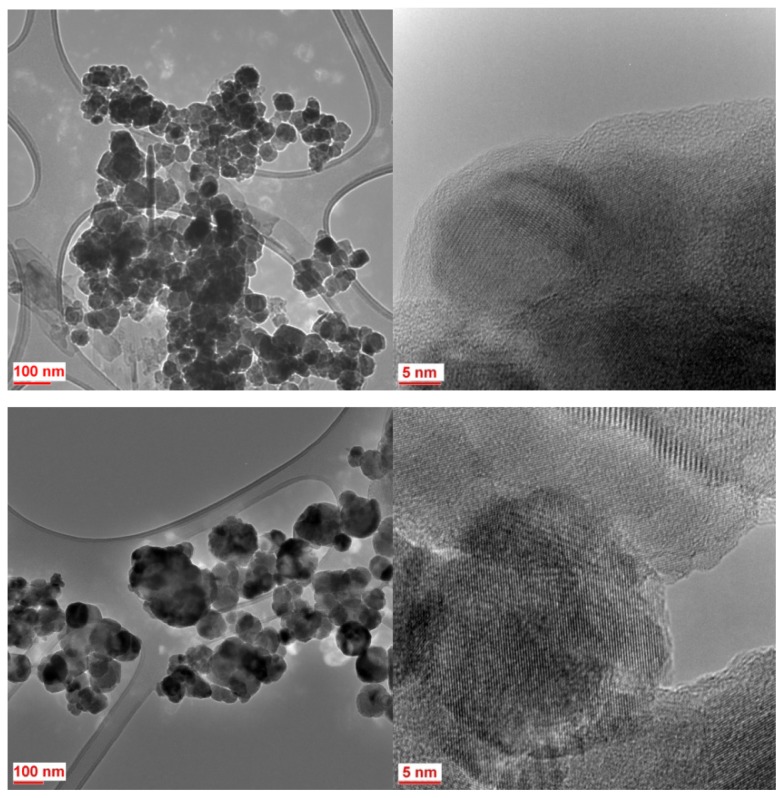
HRTEM images of two typical samples prepared at 5 V/60 °C/0.04 M concentration of Bu (**top**) and Tu (**bottom**), with different magnification.

**Figure 4:**
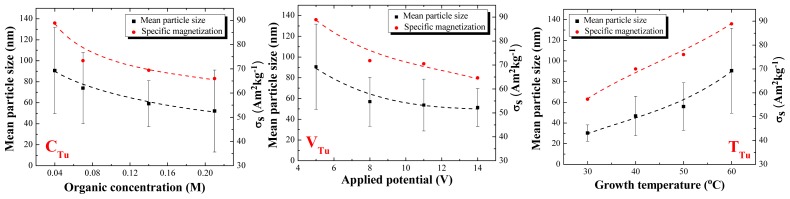

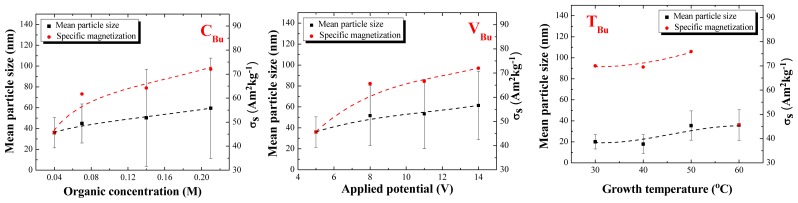
The dependence of mean particle size and σ_s_*versus* organic concentration, applied potential and growth temperature of all magnetite nanoparticles. We vary *C*_Tu_, _Bu_, with *V* and *T* at 5 V and 60 °C, *V*_Tu, Bu_, with *C* and *T* at 0.04 M and 60 °C, or *T*_Tu, Bu_, with *C* and *V* at 0.04 M and 5 V (Trends are shown by the dashed lines).

The particle size was determined using Image-J like measurement software, for at least 100 particles of each sample, based on their electron microscope images. Then the mean particle size and size distribution histograms were obtained using SPSS statistical software. Figure 2 shows a sequence of typical SEM images of magnetite nanoparticles synthesized with Tu and Bu, varying the concentration of organic agent, bath temperature and applied voltage. To the right, are shown the corresponding size distribution histograms. It can be seen that at high concentration of organics, and also at high voltage, a broad particle size distribution with some sheet-like structures mixed with agglomerated fine nanoparticles are observed. At lower growth temperature, smaller particles with a narrow size distribution were grown. Figure 3 shows TEM images of two typical samples prepared at the same experimental conditions (5 V/60 °C/0.04 M concentration of Bu or Tu) with different magnification. The crystalline magnetite nanoparticles are coated by a noncrystalline organic layer 2–3 nm thick. Figure 4 shows the mean particle size and standard deviation as a function of organic concentration, growth temperature and also applied potential for six groups of magnetite nanoparticles labeled by the concentration, temperature and voltage; for either Tu or Bu; the concentration of organic is varied, keeping *V* and *T* at 5 V and 60 °C, the temperature is varied keeping *V* and *C* at 5 V and 0.04 M, or the potential varied keeping *C* and *T* at 0.04 M and 60 °C.

By comparing the mean particle size as a function of concentration in the case of Tu, the particles synthesized at all Tu concentration are highly polydisperse. A way to reduce this polydispersity is to work at low temperature and carry out the synthesis at high overpotential. These results show that the size and morphology of electrosynthesized nanoparticles depend on the complex interplay of experimental conditions; the nature of the organic agent has a strong effect, but it is not readily predictable. The change in morphology and size with increasing additive concentration takes place because the flocculation effect of the organic influences the morphological evolution or the agglomeration of the nanoparticles. At 30 °C this effect decreases and Bu molecules cover more fully the magnetite particles, preventing them from sticking to each other which leads to the formation of smaller particles. As can be seen, there is a direct relationship between temperature and mean particle size, and it is evident that the size distribution becomes broader as temperature increases. In addition, it can be seen that by applying high voltages, the particles synthesized with Bu are not uniform and a few typical octahedral magnetite crystals are seen in some of the SEM images. Furthermore, in this case, the particle size distribution becomes broader at high voltages. This could be related to increased chemical reactivity of the organic material.

In general, concentration, voltage and temperature are all expected to drive the electrochemical reaction towards the formation of magnetite nanoparticles. At high concentration, applied voltage and temperature, the initial nuclei are more likely to cluster together, since they are all being formed at once. This leads to a smaller number of larger, more stable particles, as seen for Bu in Figure 4. The behavior of Tu is different. Tu in water is protonated and therefore, positively charged, but this charge is not permanent. After applying a voltage to the electrochemical cell, these molecules migrate towards the cathode where OH^−^ ions are being generated. The OH^−^ ultimately deprotonates Tu, which makes it less soluble. By increasing the concentration of Tu, flocculation at the cathode is enhanced, causing formation of nanoparticles with different morphology.

### 2.3. Magnetic Properties

**Figure 5:**
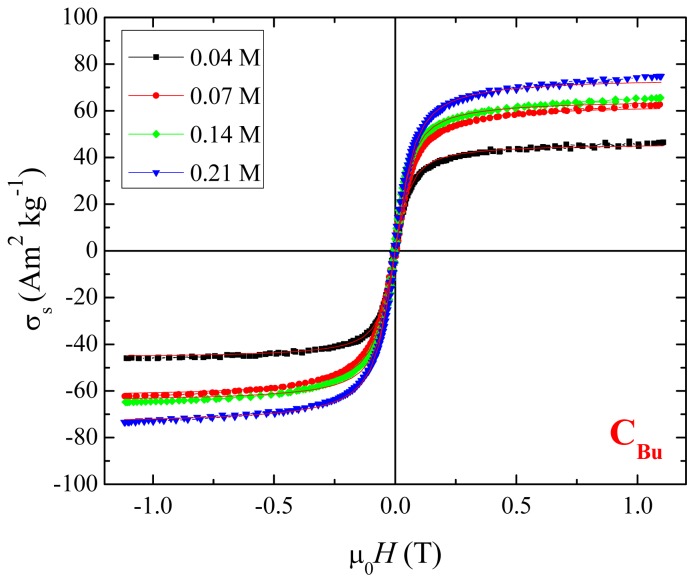
Room temperature magnetization curves of magnetite nanoparticles, belonging to the *C*_Bu_ group (*V* and *T* at 5 V and 60 °C).

Figure 4 includes a plot of specific magnetization *versus* organic concentration, applied potential and growth temperature for all six groups of magnetite nanoparticles deduced from magnetization curves like those shown in Figure 5. There, data are presented for the *C*_Bu_ group. All samples are magnetically soft with only a little hysteresis, but the specific magnetization, σ_s_ is highly dependent on the nature of the organic molecules and growth conditions.

Figure 4 demonstrates that there is a direct correlation between mean particle size and specific magnetization. The smaller particles, which have a larger surface/volume ratio, have the lower magnetization. This might be due to more oxidation at the surface, but the Mössbauer spectra do not support this idea (Section 2.4). The reduction in magnetization could therefore be attributed to canted surface spins [31], or an increased nonmagnetic organic fraction in the samples. The larger particles exhibit a σ_s_ value close to that of bulk magnetite (~90 Am^2^ kg^−1^). The only exception is the sample synthesized with 0.04 M Bu, applying 5 V at 60 °C where the lower σ_s_ value is due to the presence of a paramagnetic ferric salt formed between Bu and Fe^3+^, whose presence is evidenced by Mössbauer spectroscopy. Bu has a carboxylic group in its structure that chelates the Fe^3+^ in solution; therefore, a ferric salt is formed in the electrolyte formed at high temperature. We have observed this effect in a previous report, where we looked at 14 different agents at 60 °C, but kept the concentration and voltage constant [30]. Although some magnetite samples have similar mean particle size, they may still exhibit different specific magnetization. This discussed further in Section 2.4.

**Figure 6:**
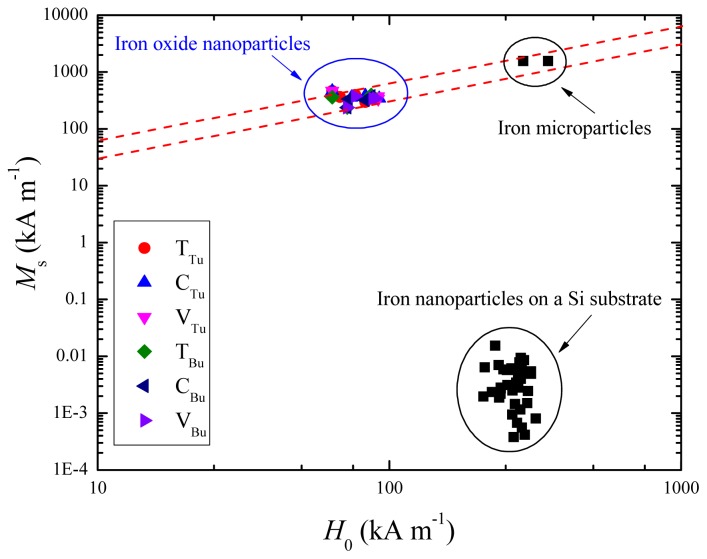
A scatter plot of *M_s_versus H*_0_ for all the magnetite nanoparticles.

None of the magnetization curves exhibited any appreciable coercivity, and they were well fitted to the empirical expression

(1) $$M=M_{s}\;\text{tanh}(H/H_{0})$$

where *M_s_* is the saturation magnetization, and *H*_0_ is an effective field that governs the approach to saturation. It is readily estimated by extrapolating the slope at the origin, which reaches the saturation magnetization when *H = H*_0_ [32]. Figure 6 shows a scatter plot of *M_s_versus H*_0_ for all iron oxide samples discussed here, with the same units of kA m^−1^ for both axes.

We include data on metallic iron microparticles and iron nanoparticles on an etched silicon substrate [33], which also exhibit characteristically anhysteretic magnetization curves with little temperature dependence below room temperature. These are the signs that the saturation of the magnetization is controlled by the magnetic dipolar field and not by magnetocrystalline anisotropy, which would normally give rise to temperature dependent coercivity at low temperature. If we assume a uniform demagnetizing field

(2) $$H_{\text{d}}=-NM$$

where *N* is the demagnetizing factor, the two dashed lines in the Figure 5 show the expected trends for *N* = 1/3 and *N* = 1/6. The iron nanoparticles on silicon fall in a quite different area, because only a tiny fraction ~10^−5^ of the sample volume is magnetic. It is likely that our magnetite particles have a vortex structure [34] for which *N*_eff_ ≈ 0.2.

### 2.4. Mössbauer Spectroscopy

**Figure 7:**
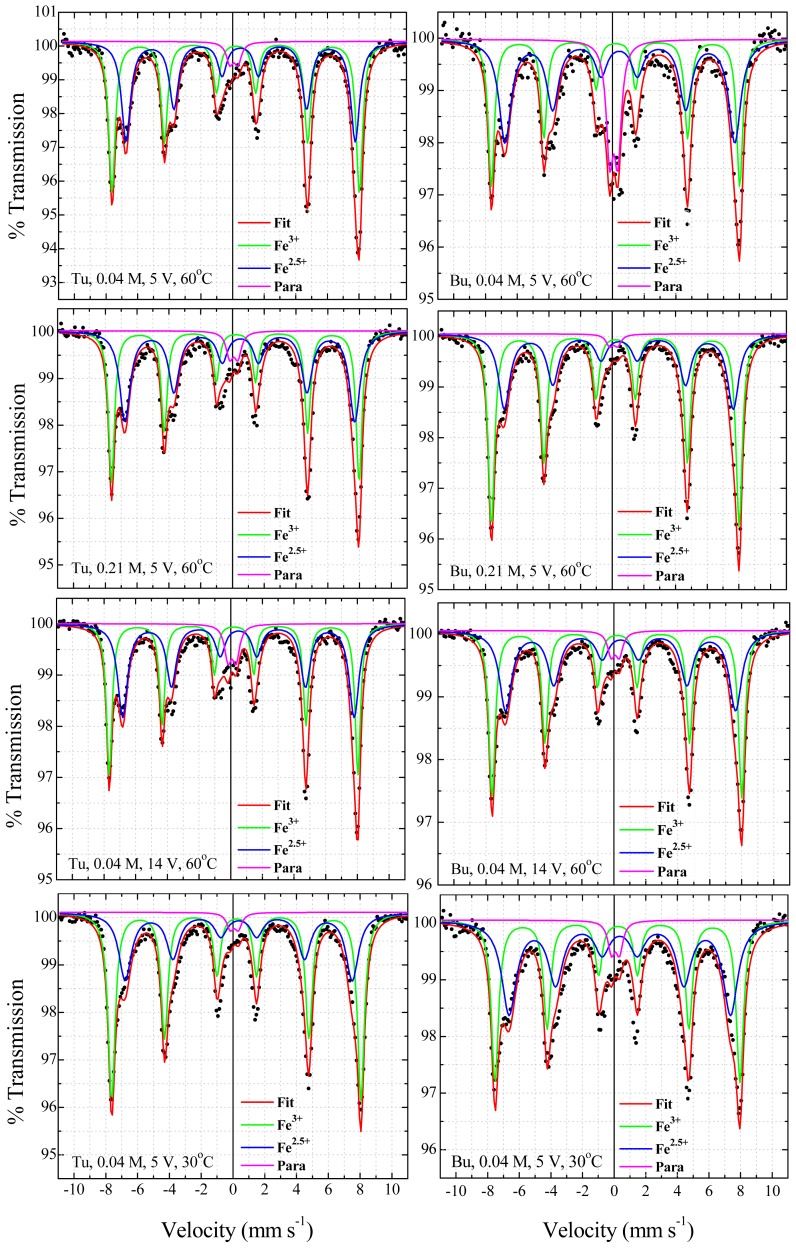
Experimental and fitted room-temperature Mössbauer spectra for magnetite nanoparticles prepared at different growth condition.

**Figure 8:**
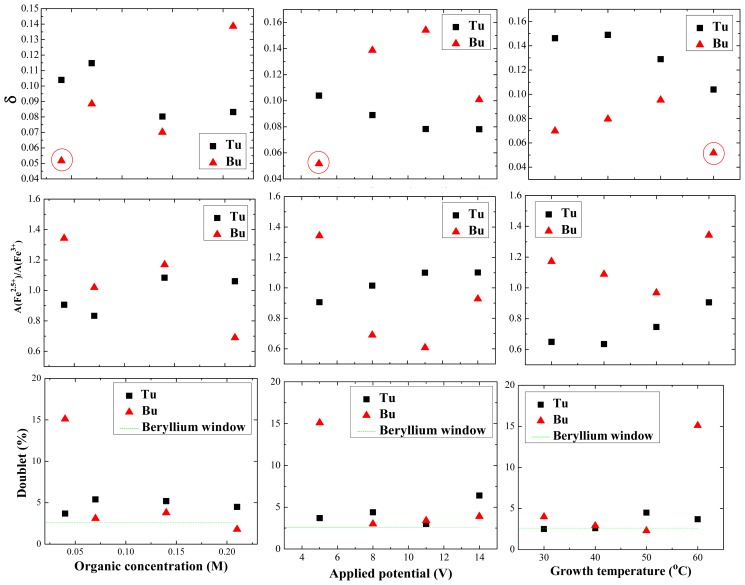
The dependence of the paramagnetic doublet contribution, the ratio of Fe^2.5+^ to Fe^3+^ and the nonstoichiometry parameter δ as a function of growth parameters for all samples (**left column**, *V* and *T* at 5 V and 60 °C, middle column, *C* and *T* at 0.04 M and 60 °C; **right column**, *C* and *V* at 0.04 M and 5 V). The circled point refers to the sample with an secondary iron phase.

Figure 7 shows the experimental and fitted room-temperature Mössbauer spectra for magnetite nanoparticles prepared under different growth conditions, in the presence of Tu and Bu. The spectra are well fitted by two magnetic sextets associated with magnetite, plus a central paramagnetic doublet, which accounts for the presence of a paramagnetic secondary phase associated with organometallic complexes containing Fe^3+^ and Bu. It rapidly decreases in intensity by increasing the Bu concentration and applied voltage or decreasing the temperature. Another weak paramagnetic doublet with isomer shift 0.09 mm s^−1^ and quadrupole splitting 0.35 mm s^−1^ which is present in all the spectra is an artifact associated with iron in the beryllium window of the proportional counter; it corresponds to only about 3% of the total absorption (shown with a green dashed line in Figure 8). The dependence of the paramagnetic doublet contribution, the ratio of Fe^2.5+^ to Fe^3+^ and the nonstoichiometry parameter δ in the formula Fe_3−δ_O_4_ as a function of growth parameters for all samples deduced from the intensity ratio of the two magnetite subspectra are presented in Figure 8.

Magnetite can exhibit a range of stoichiometry with a deficit of iron, and the extremes are δ = 0 (stoichiometric magnetite) and δ = 0.67(γ-Fe_2_O_3_) in the formula Fe_3−δ_O_4_. In stoichiometric magnetite, the iron in octahedral coordination (B-site) has an average electronic configuration of Fe^2.5+^, which arises from fast electron hopping among the iron on the octahedrally-coordinated sites. Hence, the ratio of the areas of the Fe^3+^ (A-site) to Fe^2.5+^ (B-site) spectra is expected to be 1:2. For non-stoichiometric magnetite, the vacancies are on B-sites, and the charge balance is preserved by a greater proportion of Fe^3+^ ions there. The two six-line subspectra are not attributed simply to A and B site iron, but to iron in an Fe^3+^ configuration (A-site and some B-site iron) and iron in an Fe^2.5+^ configuration involved in electron hopping (the remaining B-site iron) [30,35]. For Fe^3+^ and Fe^2.5+^, isomer shifts are 0.22 ± 0.02 and 0.45 ± 0.05 mm/s, line width are 0.24 ± 0.04 and 0.45 ± 0.11 mm/s, quadrupole shifts are 0.02 ± 0.02 and 0.25 ± 0.25 mm/s, and hyperfine fields are 48.5 ± 0.5 and 44.4 ± 0.9 T, respectively. For all samples, the ratio of Fe^2.5+^ to Fe^3+^ is presented in Figure 8 together with the iron deficit δ. For the sample produced in the presence of 0.04 M Bu, applying 5 V at 60 °C, δ is 0.05. It changes to 0.14, 0.10 and 0.07, by changing the Bu concentration, applied voltage or growth temperature to 0.21 M, 14 V and 30 °C, respectively. Also in the case of Tu, this parameter is 0.10 for the sample prepared with 0.04 M Tu, applying 5 V at 60 °C and it changes to 0.08, 0.08 and 0.15 by changing the Tu concentration, applied voltage or growth temperature to 0.21 M, 14 V and 30 °C, respectively. Based on these results, the sample prepared in the presence of Bu, with chemical formula Fe_2.95_O_4_ is closer to stoichiometric magnetite than any of the others.

## 3. Experimental Section

### 3.1. Materials

Thiourea (Tu) and sodium butanoate (Bu), were purchased from Sigma-Aldrich Chemical Co. Sodium sulfate anhydrous supplied by BDH limited Poole England and iron sheet (purity 99.5%) was purchased from Advent Research Materials.

### 3.2. Methods

Magnetite nanoparticles were synthesized by electro-crystallization of iron using a chronoamperometric technique in the presence of an organic stabilizer from an aqueous medium [26,27]. Two purified iron plates of 1 × 4 cm^2^ and 1 × 1 cm^2^ were used as cathode and anode, respectively. The electrodes were polished with fine-grain emery paper and ultrasonically cleaned with ethanol. Two cleaned electrodes were placed a distance of 1 cm apart from each other in an electrochemical cell containing a solution of one of two different organic additives: Tu and Bu, with 0.25 M sodium sulfate anhydrous salt as the electrolyte. At the beginning of the experiment when the potential was imposed, the solution was colorless. After applying an appropriate potential difference using a Solartron Instruments SI 1280B electrochemical measurement unit, water is reduced to hydrogen and hydroxyl anions at the cathode and the iron anode is oxidized to Fe^2+^ and Fe^3+^. Therefore in the solution, Fe^2+^, Fe^3+^ and OH^−^ meet to react and produce an orange-brown iron hydroxide, which then dehydrates to form a black magnetite, (Fe_3_O_4_) precipitate. The reaction time was chosen as 30 min and the electrolyte temperature was controlled using a thermostatic water bath. The black precipitates were separated from the reaction medium using a Nd-Fe-B permanent magnet and washed several times with a copious amount of DI water and let dry.

In order to investigate the effect of organic additive concentration, applied potential, and growth temperature on structure and particle magnetic properties, six different series of samples named *C*_Tu_, *C*_Bu_, (0.04, 0.07, 0.14, 0.21 M), and *V*_Tu_, *V*_Bu_ (5, 8, 11, 14 V), as well as *T*_Tu_, *T*_Bu_, (30, 40, 50, 60 °C) were synthesized in the presence of either Tu or Bu as organic additive. For each organic, samples were first synthesized at four different concentrations. The 0.04 M concentration was used at different voltages, and finally 0.04 M and 5 V materials were synthesized at different temperatures.

The crystal structure of the products was determined by a Philips X-ray diffractometer, using CuK_α_ radiation (λ = 1.5405 Å) generated at 40 kV and 40 mA. An FEI Titan high resolution transmission electron microscope (HRTEM) and a Carl Zeiss Ultra Plus scanning electron microscope (SEM) were employed to investigate the particle size, morphology, and nanostructure of the magnetite particles. Magnetic measurements were carried out using a home-made vibrating-sample magnetometer (VSM) with a 1.1 T permanent magnet flux source. Mössbauer spectra in the transmission geometry were recorded for all samples using a Co^57^ source in Rh. Isomer shifts are quoted relative to the source.

## 4. Conclusions

The results of our study on the effect of the experimental conditions on structural and magnetic properties of electrocrystallized magnetite nanoparticles in the presence of different organic molecules show that temperature, voltage and concentration all play a significant role in determining the morphological, structural, and magnetic properties of the nanoparticles produced. The particle size and morphology were found to be easily modified by the concentration of organic agent, applied potential, or the temperature of the bath. Magnetometry showed that the specific magnetization value of the Fe_3_O_4_ nanoparticles ranges from 45 to 90 Am^2^ kg^−1^, depending on the type of organic molecules and experimental conditions, but much of this variation is actually due to the mass of attached, nonmagnetic organic material rather than any change in stoichiometry of the Fe_3−δ_O_4_. Analysis of Mössbauer spectra showed that by tuning the growth parameters, it is possible to effectively remove a paramagnetic phase, associated with organometallic complexes containing Fe^3+^ which were present in samples prepared with Bu. The iron is then exclusively present in the form of slightly nonstoichiometric magnetite, with nonstoichiometry parameter δ ≈ 0.1.
